# Indicated prevention interventions for anxiety in children and adolescents: a review and meta-analysis of school-based programs

**DOI:** 10.1007/s00787-020-01564-x

**Published:** 2020-06-13

**Authors:** Siobhan Hugh-Jones, Sophie Beckett, Ella Tumelty, Pavan Mallikarjun

**Affiliations:** 1 School of Psychology, University of Leeds, Leeds, LS2 9JT UK; 2 The Barberry National Centre for Mental Health, University of Birmingham, Birmingham, B15 2FG UK

**Keywords:** Child, Adolescent, School-based interventions, Anxiety, Early intervention, Meta-analysis

## Abstract

Anxiety disorders are among the most common youth mental health disorders. Early intervention can reduce elevated anxiety symptoms. School-based interventions exist but it is unclear how effective targeted approaches are for reducing symptoms of anxiety. This review and meta-analysis aimed to determine the effectiveness of school-based indicated interventions for symptomatic children and adolescents. The study was registered with PROSPERO [CRD42018087628]. We searched MEDLINE, EMBASE, PsycINFO, and the Cochrane Library for randomised-controlled trials comparing indicated programs for child and adolescent (5–18 years) anxiety to active or inactive control groups. Data were extracted from papers up to December 2019. The primary outcome was efficacy (mean change in anxiety symptom scores). Sub-group and sensitivity analyses explored intervention intensity and control type. We identified 20 studies with 2076 participants. Eighteen studies were suitable for meta-analysis. A small positive effect was found for indicated programs compared to controls on self-reported anxiety symptoms at post-test (*g* = − 0.28, CI = − 0.50, − 0.05, *k* = 18). This benefit was maintained at 6 (*g* = − 0.35, CI = − 0.58, − 0.13, *k* = 9) and 12 months (*g* = − 0.24, CI = − 0.48, 0.00, *k* = 4). Based on two studies, > 12 month effects were very small (*g* = − 0.01, CI = − 0.38, 0.36). No differences were found based on intervention intensity or control type. Risk of bias and variability between studies was high (*I*^2^ = 78%). Findings show that school-based indicated programs for child and adolescent anxiety can produce small beneficial effects, enduring for up to 12 months. Future studies should include long-term diagnostic assessments.

**Electronic supplementary material:**

The online version of this article (10.1007/s00787-020-01564-x) contains supplementary material, which is available to authorized users.

## Introduction

Globally, approximately 117 million children and young people are affected by anxiety disorders [1]. Fewer than 20% of young people with anxiety disorder access support [2], and of those that do, a significant minority end treatment prematurely or do not benefit [3, 4]. Cost-effectiveness analyses indicate that treatment alone is insufficient to eliminate the disease burden of these disorders and that investment in prevention and early intervention are needed [5]. Evidence-based prevention, early intervention and treatment approaches exist for anxiety [6] with broadly similar content, only differing substantively in terms of the time point at which they are delivered [7]. Elevated anxiety symptoms affect quality of life and are a risk factor for anxiety disorders. The reduction of elevated symptoms is therefore important to improve functioning and well-being, and to potentially prevent new cases of disorder. Schools are considered good sites for early intervention programs given their reach and circumventing of common barriers to support [8]. Programs can be universal (delivered to all pupils) or targeted, being either selective (delivered to those at higher risk, based, for example, on family history), or indicated (delivered to those with detectable but sub-clinical symptoms) [9]. Most studies evaluate interventions in terms of their impact on anxiety symptoms rather than diagnostic outcomes, possibly due to the resource requirements for diagnostic assessments. It is unclear which delivery approach is most effective for reducing symptoms of anxiety and which should be implemented in schools.

Meta-analyses have reviewed the effects of different delivery formats. Interpretations of the value of reported effect sizes (ES) from this body of work need to consider a number of factors [10]. For example, the importance of an effect can depend on intervention costs, ease of delivery, scale-up penalties, school attendance and academic/social benefits, changes in health care utilisation, numbers of new cases averted and spill over effects to peers [10]. Small ES from relatively inexpensive, scalable interventions (e.g., in schools where the infrastructure exists), that reach large sections of the population at a time of vulnerability to mental health disorder could have substantial practical importance, considering the high prevalence of anxiety, its psychosocial and economic impact, and that so few young people access support via other means [2].

Most school-based anxiety interventions to date have been universal and highly heterogeneous. Meta-analyses of these report small-to-moderate post-test ES in the range of 0.2–0.5 on anxiety symptoms in children and adolescents [11–20]. Although 12 month effects from universal programs have been reported for depressive symptoms [10, 21], similar outcomes from universal programs for anxiety symptoms are rare, with only a few studies reporting ES spanning small, marginal, or no effects at 12 and/or 24 months [12, 14–17, 19, 20]. Targeted approaches for depression, delivered in school to at-risk young people, appear more effective than universal approaches, suggesting that intervention effectiveness increases with symptom severity [21, 22] However, findings from studies of targeted approaches to reduce anxiety symptoms are mixed. Lawrence, Rooke, and Creswell’s (2017) meta-analysis of 16 RCTs, which merged indicated and selective trials, reported a significant small-to-moderate effects (ES = − 0.43) on post-test anxiety symptoms compared to waitlist controls (*k* = 10) but only a very small non-significant effect compared to attention controls (ES = − 0.09, *k* = 5) [15]. Effects increased up to 6 months but fell at 12 and 24 months.

Two reviews have compared universal and targeted program effectiveness. Merging studies of indicated and selective interventions, Teubert and Pinquart’s (2011) review of 65 trials (where anxiety symptoms or diagnostic status was a primary or secondary outcome) found that, at post-test, targeted programs (*k* = 36 studies) were associated with a significantly higher mean ES (0.32) than universal programs (0.12), although this difference was no longer significant at follow-up (0.15 for universal and 0.23 for indicated) (0.23) [17]. In comparison, Werner-Seidler et al.’s (2017) review of 81 original randomised-controlled trials (RCTs), which also merged indicated and selective programs, found no significant differences in ES between these and universal programs for anxiety symptoms across all ages in the short, medium, or long terms [20]. Some targeted approaches performed no better than universal approaches in the short term or long term. In sum, reviews have produced mostly small post-test ES for targeted approaches. However, the merging of selective and indicated trials in these reviews is problematic as the former targets asymptomatic young people and the latter targets those with detectable elevated symptoms. Indicated interventions have the potential to be more effective than selective interventions given the scope for change in symptoms, and the targeting of especially responsive groups who are motivated to engage in the intervention [10]. To date, only one review has isolated the effect of school indicated interventions for reducing symptoms of anxiety. Neil and Christensen’s (2009) review of 27 trials (published between 1987 and 2008) spanned universal (*k* = 16), indicated (*k* = 8), and selective (*k* = 3) programs for 5–19 year olds [16]. At post-test, 69% of the universal programs (*k* = 11) reported significant differences between the intervention and control conditions at post-test, with moderate-to-very large ES (0.31–1.37). This compared to only 50% of indicated trials (*k* = 4) with small-to-moderate ES (0.20–0.76). At follow-up, however, indicated trials outperformed universal programs. Out of six indicated trials that reported follow-up data (spanning 1–30 months), all but one (83%) reported significant small-to-large effects (0.19–1.03). Out of the six trials of universal programs with follow-up data, 3 (50%) reported significant small-to-moderate effects (0.22–0.70). Thus, compared to universal programs, a higher proportion of indicated programs secured long-term symptom reduction, and some with larger effects (although the difference was not formally tested). However, the number of indicated trials was low and the quality poor.

A number of new trials of school indicated approaches for reducing anxiety symptoms have been conducted since Neil and Christensen [16]. Producing an updated ES for indicated school programs will progress the evidence for effective early intervention strategies for symptom reduction that can be delivered at a population level. The aim of our review was to (1) synthesise evidence on school indicated programs for reducing elevated (sub-clinical) anxiety symptoms in children and adolescents; (2) conduct a meta-analysis to identify their overall effects; (3) establish the duration of any effects; and (4) determine the relative effect of interventions according to control type, delivery agent, and intervention intensity.

## Methods

### Data sources and searches

We searched MEDLINE, EMBASE, PsycINFO, and the Cochrane Central Register of Controlled Trials, ClinicalTrials.gov, and PROSPERO from database inception to December 2019. Details of search strategies are provided in Suppl. Table 1. Grey literature was searched using Google Scholar. Reference lists of relevant studies and reviews were searched. Study authors were contacted in the case of missing or ambiguous information. The review protocol was developed using the Preferred Reporting Items for Systematic Reviews and Meta-analyses (PRISMA) statement guidelines [23] and was registered with PROSPERO [CRD42018087628 https://www.crd.york.ac.uk/prospero/display_record.php?RecordID=87628].

### Study selection

Studies were eligible for this review if they were published in English and met the following PICOS inclusion criteria: *(P) Population* children/adolescents aged 5–18 years with elevated levels of anxiety. Studies with both sub-clinical and (likely or confirmed) clinical populations were included only where the intervention aim was described as preventative rather than treatment. This decision accommodated any small variability in screening or diagnostic outcomes within a study, and the fact that many studies reported only symptom thresholds for inclusion and not upper thresholds for exclusion.

*(I) Intervention* school-based indicated prevention or early intervention programs for anxiety disorders, where school-based means endorsed by schools and delivered on school premises either face-to-face or online*.* Multi-tiered interventions were included if they contained an indicated intervention. Although our published protocol stated that we would include interventions that targeted depression and anxiety, Werner-Siedler et al. [20] recently reviewed indicated programmes for depression; hence, we then restricted our review to interventions for anxiety. *(C) Comparator* passive comparators (waitlist and no intervention) and active comparators (programs to control for non-specific aspects of anxiety treatment). *(O) Outcome* anxiety symptoms measured via validated self-rating or clinician-rated scales and/or diagnostic outcomes (i.e., no longer meeting diagnostic thresholds showing symptom reduction). *(S) Study design* randomised-controlled trials.

### Outcome measures

Our main outcome was reduction of anxiety symptoms and prevention of anxiety disorder progression, i.e., a change in symptoms that could represent prevention of disorder. Our primary outcome for efficacy was the mean change scores on anxiety symptoms in comparison to controls, based on self-rating or clinician-rated scales. Table 1 shows the primary outcome measures utilised in each study and included the Revised Children’s Anxiety and Depression scale [24], the Spence Children’s Anxiety Scale [25], the Revised Children’s Manifest Anxiety Scale [26], the Screen for Child Anxiety Related Disorders [27], the State-Trait Anxiety Inventory [28], the Multidimensional Anxiety Scale for Children [29], the Child Behaviour Checklist [30], and the Social Phobia subscale [31]. Where a trial included more than one scale for anxiety symptoms, we used the scale with the greatest frequency across studies. Change in anxiety symptoms was also assessed in some studies via diagnostic interviews.

**Table 1:** Characteristics of included trials (*k* = 20) by date of publication

Trial citation	Program	*N* Age (years)	Control	Delivery	Content	Primary anxiety measure	Post-test effect size*	Follow-up effect size (months)
Kiselica et al. [41]	Stress inoculation	48 14–15	NI	MHP + school MHP	CBT	STAI A-TRAIT	− 0.74^a^	− 1.01 (1)^c^
Dadds et al. [36]	Coping koala	128 7–14	NI	MHP + Grad	CBT	RCMAS	0.01	− 0.05 (6)
Mifsud and Rapee [51]	Cool kids	91 8–11	WL	MHP + school MHP	CBT	SCAS	− 0.35	− 0.57 (4)^c^
Bernstein et al. [34, 54]	FRIENDS (child + parent)	61 7–11	FRIENDS child only + WL	MHP + Grad	CBT	MASC^b^	0.22	− − 0.04 (6)
Gillham et al. [39]	Penn resiliency program	44 11–13	NI	Grad/ researchers	CBT	RCMAS	− 0.07	− 0.62 (6)^c^ − 0.79 (12)^c^
Siu [47]	FRIENDS	47 7–10	WL	MHP	CBT	SCARED	− 1.48^a^	NA
Hunt et al. [38]^a^	FRIENDS	260 11–13	NI	School MHP + Teacher	CBT	SCAS	No control group data	0.17 (24) − 0.01 (48)
Siu [48]	Theraplay	46 Mean 7.8	WL	MHP	Play therapy	Internalising scale of CBCL	− 2.40^a^	NA
Manassis et al. [37]	Feelings club	148 8–12	AC	MHP + Grad	CBT	MASC^b^	− 0.06	− 0.06 (12)
Liddle and Macmillan [43]^a^	FRIENDS	51 8–14	WL	MHP	CBT	SCAS	No control group data	NA
Cooley-Strickland et al. [35]	FRIENDS	93 8–12	WL	MHP + Grad	CBT	RCMAS	0.21	NA
Miller et al. [52]	FRIENDS	191 9–12	AC	Teacher + MHP/Grad	CBT	MASC	0.08	NA
Nobel et al. [45]	Feelings Club	78 8–11	AC	MHP	CBT	MASC	0.03	NA
McLoone and Rapee [44]	Cool Kids	152 7–12	Home + WL	School MHP	CBT	SCAS	− 0.43	− 0.27 (12)
Sportel et al. [33]; de Hullu et al. [55]	Cognitive Behavioural Group	240 12–16	CBT + NI	MHP	CBT	SP subscale of RCADS^b^	0.16	− 0.41 (6)^c^ − 0.17 (12) − 0.21 (24)
Yulei et al. [50]	Cool Kids	59 14–17	WL	MHP + Grad	CBT	SCAS	− 0.45	NA
Hadwin et al. [40]	Cogmed-WM	40 11–14	AC	Computerised	WM training	RCMAS	− 0.46	0.00 (3)
Lam [42]	MBCT-C	20 9–13	WL	MHP + Grad	Mindfulness	RCADS	0.22	NA
Scholten et al. [46]	Dojo video game	138 11–15	AC	Computerised + researcher	ERT + HRV biofeedback	SCAS	− 0.11	0.03 (3)
Van Starrenburg et al. [49]	Coping Cat	141 7–13	WL	MHP + Grad	CBT	SCAS	− 0.58^a^	− 0.64 (3)^c^

Mode of delivery: *MHP* Mental health professional, *Grad* graduate students. Program content: *CBT* Cognitive behavioural therapy, *MBCT-C* mindfulness-based cognitive therapy for children, *WM* working memory, *ERT + HRV* emotion regulation training and heart rate variability. Control: *WL* wait list, *NI* no intervention, *AC* attention control. Anxiety outcome measures: *RCADS* Revised Children’s Anxiety and Depression scale, *SCAS* Spence Children’s Anxiety Scale, *RCMAS* Revised Children’s Manifest Anxiety Scale, *SCARED* The Screen for Child Anxiety-Related Disorders), *STAI A-TRAIT* State-Trait Anxiety Inventory, *MAS* Multidimensional Anxiety Scale for Children, *CBCL* Child Behaviour Checklist, *SP subscale* Social Phobia subscale. Follow-up effect sizes: *NA *not available (typically as waitlist controls had started the intervention by follow-up, so no true control group data available)

*Negative effect sizes indicate reduction in symptoms

^a^Excluded from the meta-analysis

^b^Also administered post-intervention diagnostic interviews

^c^Significant differences in anxiety scores between intervention and control groups

### Screening, data extraction, and quality assessment

Three authors (SB and PM) and a medical student (independent of study) separately screened the titles and abstracts of studies against the inclusion criteria in the first round of searching (up to September 2018). In the updated search (up to December 2019), abstracts were separately screened by two authors (PM, ET). Independent full-text reviews were completed by SB and PM (first search) and ET, PM and SHJ (updated search). Studies were included if they met all of the inclusion criteria. Disagreements between reviewers were resolved through discussion. For the included studies, data were extracted, using a Cochrane data abstraction form, by one investigator (SB) and were independently checked by another (PM). Data extracted included study details (author, publication year, and country), participant details (age range, gender, sample size, and school type), methodology (design, unit of allocation), intervention characteristics (type, content, target, provider, parental involvement, frequency, duration, and indicators of acceptability), control group (type), and all reported outcomes (definition, time points, person measuring, summary estimates, fidelity, acceptability, and compliance). For the waitlist control studies, follow-up data were only extracted if the waitlist control group remained waitlist at follow-up. Risk of bias was assessed with the Cochrane Handbook Risk of Bias Assessment Tool [32]. Risk of bias was assessed independently by one investigator (SB) and a medical student, neither of whom were blinded to the review aim. As recommended by Cochrane, risk of bias was reported separately for each of the seven criteria, namely random sequence generation, adequate concealment of this sequence, blinding of participants/personnel, blinding of outcome assessment, reporting of incomplete outcome data, selective reporting of data, and protection against contamination. All domains were scored as (1) low risk of bias, (2) unclear, or (3) high risk of bias. Disagreement was resolved through discussion. Study quality was rated high risk study where three or more criteria showed high risk of bias, as low risk where five or more items rates showed low risk of bias, and as moderate risk in all remaining situations.

### Statistical analysis

A meta-analysis using a random-effects model was conducted (using the RevMan software version 5.3) as considerable heterogeneity was expected across studies. The inverse variance method was used to weight studies. Continuous outcomes are presented as standardised ES. Standardised ES estimates, referring to the difference between the intervention and control group at post-intervention and follow-up, were calculated using Hedge’s *g* (with 95% confidence intervals), which includes an adjustment for small-sample sizes. Negative ES indicate a decrease, and positive ES indicate an increase, in mean levels of anxiety symptoms between time points of comparison. The *I*^2^ statistic was used to test for homogeneity of ES by indicating heterogeneity in percentages, with 25%, 50%, and 75% representing low, moderate, and high heterogeneity, respectively. Sub-group analyses were conducted for delivery agent, control type, and intervention intensity. Sensitivity analyses were conducted using only studies with a low risk of bias. A funnel plot was used to assess for small-study effects and publication bias. Egger’s tests were conducted where asymmetry was apparent on visual inspection. Duval and Tweedie’s trim-and-fill procedure was conducted using the Comprehensive Meta-Analysis software (version 3.0 Biostat Inc.) to give an adjusted estimate of unbiased ES.

## Results

### Study selection and characteristics

**Fig. 1:**
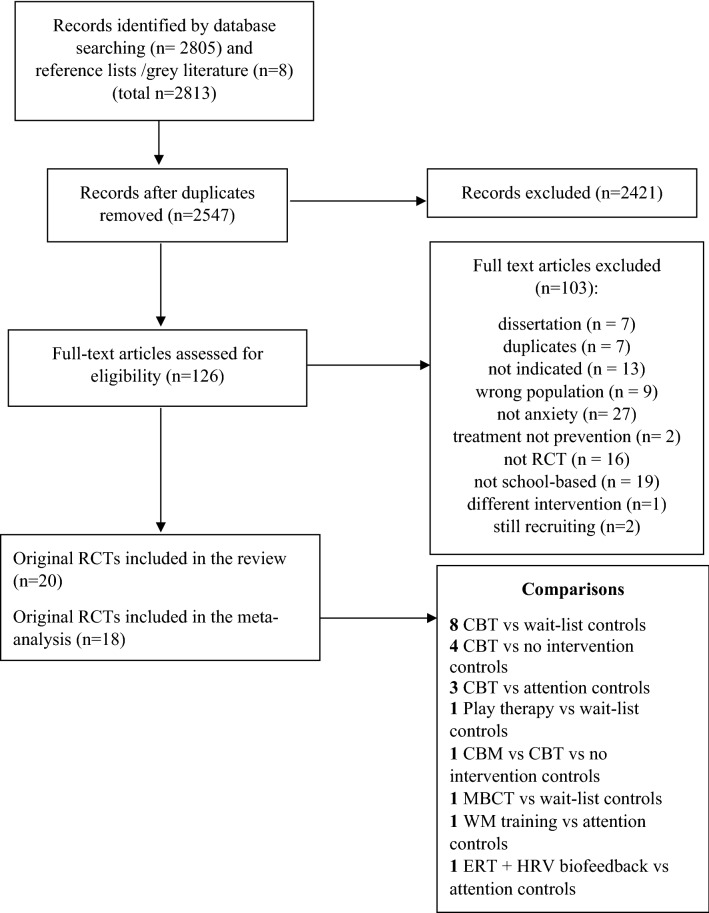
Flow diagram of selection of studies for inclusion. *CBT* cognitive behavioural therapy, *CBM* cognitive bias modification, *MBCT-C* mindfulness-based cognitive therapy for children, *WM* working memory, *ERT + HRV* emotion regulation training and heart rate variability

The PRISMA flow diagram (see Fig. 1) details the study-selection process. In total, 2813 studies were identified, of which 266 were duplicates and removed, leaving 2547 studies for title and abstract screening, following of which 2421 studies were excluded leaving 126 studies for full-text screening. Of these, 103 were excluded. Reasons for exclusion were dissertations; duplicates; not an indicated intervention; wrong population; not anxiety; treatment not prevention; not RCT; not school-based; different intervention; and still recruiting. This left 23 studies for inclusion, of which 20 were original studies.

Characteristics of study samples, intervention, delivery agent, control type, and primary anxiety measure are shown in Table 1. The 20 reviewed studies included 2076 participants. All studies targeted generalised or unspecified anxiety except for one [33] which focused on social and test anxiety. Four studies (20%) used diagnostic screening interviews that led to the inclusion of children who met both clinical and sub-clinical thresholds for anxiety disorder [33–36]. We included these as the interventions still had a preventive purpose for those not meeting clinical thresholds. The remaining 16 studies (80%) used self, parent, or teacher report to screen for baseline elevated anxiety symptoms. Screening tools and cut-off scores used to determine inclusion varied across studies. Whilst most studies utilised standardized measures at outcome, six (30%) also utilised diagnostic interviews [33–38].

Studies randomised participants at the individual (*k* = 13; 65%) [35, 39–50], group (*k* = 1; 5%) [37], and school levels (*k* = 6; 30%) [33, 34, 36, 38, 51, 52]. With one exception (which was computerised) [46], all programs were delivered to groups, and program sessions (ranging from 40 to 90 min) were usually conducted weekly (*k* = 17; 85%) [34, 36–45, 47–52], also twice weekly (*k* = 2; 10%) [33], [46] or biweekly (*k* = 1; 5%) [35]. Only two programs included booster sessions.[34, 38] One study delivered a booster package of intervention prompts to the families of the intervention group children post-intervention [36]. Secondary outcomes included academic achievement [35][40, 41]and two studies also reported on intervention acceptability.[42, 48] Of the 19 programs delivered face-to-face, only seven (37%) were subjected to fidelity assessments, using either independent ratings of audio or video recordings of the sessions (*k* = 4; 21%) [37, 38, 42, 52] or had supervised sessions (*k* = 3; 16%). [34, 36, 49] Of the ten studies (50%) that reported on compliance [33–36, 39, 40, 42, 44, 47, 51], all reported high completion rates with at least more than 50% of participants attending more than 50% of the sessions. Drop out during the intervention was only clearly reported in 12 studies, and ranged from no dropout to a drop out of 35% [32] (see Suppl. Table 1) for further study details including screening measures, nature of control conditions, number of parent and child sessions, and other indicators of compliance (session attendance and measure non-completion).

The risk of bias was high, although most studies had an unclear risk of selection bias due to inadequate reporting of the randomisation procedure. Risk of contamination was high in 14 studies (70%) (students randomised to groups within schools) and low in six studies (30%) (cluster randomisation). Blinding of participants and personnel was difficult as most studies had a wait list or no intervention control group (*k* = 15; 75%). Blinding of outcome assessment was not feasible in 14 studies (70%) as self and parent report outcome measures were used. Studies which included an outcomes diagnostic interview generally had a low risk of detection bias as interviewers were blind to the participants’ group (*k* = 4; 20%). For attrition bias, the risk was generally low. Nearly all studies had an unclear risk of reporting bias. The inter-rater reliability between the two independent reviewers for each risk of bias domain was adequate (Cohen’s Kappa > 0.80 for all risk of bias domains) (see Suppl. Fig. 1).

A meta-analysis was conducted on the 18 studies with sufficient data to compare post-test differences in anxiety symptoms between intervention and control groups (Liddle and Macmillan [43] and Hunt et al. [38]were excluded due to inadequate data). Three studies had more than one comparison group. Therefore, for McLoone and Rapee [44], the school-based not home-based treatment group was included; for Bernstein, Layne, Egan, and Tennison [34], the child-only group was combined with the child + parent group to create a collapsed treatment group; and for Sportel, de Hullu, de Jong, and Nauta [33], we compared the CBT and no intervention control group, and for Hadwin et al. (2016) [40], we compared CBT (active control) and working memory training (intervention).

### Efficacy outcomes

The overall post-test ES for change in anxiety symptoms was *g* = − 0.28 (CI = − 0.50, − 0.05) with large heterogeneity (*I*^2^ = 78%) (*k* = 18). The ES for change in anxiety symptoms in the short term (0–6 months) was *g* = − 0.35 (CI = − 0.58, − 0.13) (*k* = 9), in the medium term (6–12 months) was g = − 0.24 (CI = − 0.48, 0.00) (*k* = 4) and in the long term (> 12 months) was *g* = − 0.01, CI = − 0.38, 0.36) (*k* = 2). Significant post-test differences between intervention and control groups were reported in 4 out of 18 studies (22%) [41, 47–49] with medium-to-very large ES (based on Cohen [53]). At follow-up, significant medium-to-very large effects [10] were reported in 5 out of 11 studies with suitable data (46%).[33, 39, 41, 49, 51] Five of the eighteen trials (28%) reported small non-significant effects at either post-test or follow-up [34, 40, 44, 50][55]. Four trials (22%) failed to find any effect at either post-test or follow-up [34, 43, 44, 54]. Findings from the four studies [35, 42, 45, 52] (and their follow-up publications) that reported post-test diagnostic interviews at 12 and 24 m showed that the odds of having an anxiety disorder were significantly lower in the intervention compared to the control group at 24 m (*k* = 2, OR = 0.40, 95% CI = 0.19, 0.83) but not at 12 m (*k* = 2, OR = 0.64, 95% CI = 0.34, 1.18).

### Sub-group analyses

**Fig. 2:**
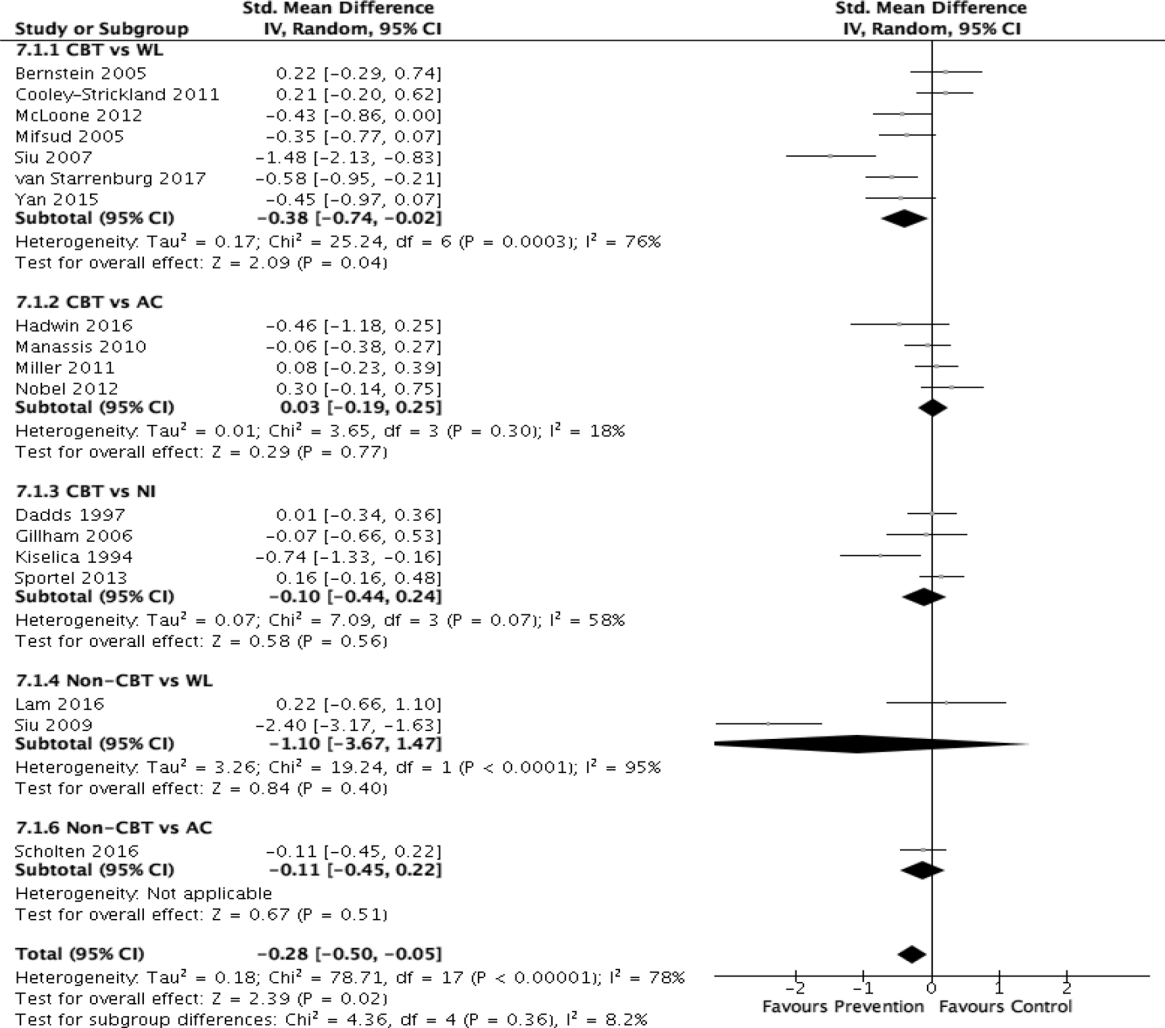
Forest plot of effect sizes for comparisons between intervention (CBT or non CBT) and control conditions (waitlist, attention control, or no intervention) on post-intervention anxiety symptoms. The vertical line indicates the line of no effect, the horizontal lines indicates the 95% confidence intervals, and the green dots represent the effect estimates from individual studies. Black diamonds indicate the pooled results of the studies

We could not perform a sub-group analysis on age (child vs adolescent) due to an insufficient number of studies that included children only (*k* = 4) and adolescents only (*k* = 3). A sub-group analysis could also not be performed on delivery agent (as very few interventions were delivered by teachers, *k* = 2) nor on program type (most were CBT based). Figure 2 depicts a Forest plot of the ES for sub-group comparisons between intervention types and control conditions on post-test anxiety scores. The only pooled studies with significant, small effects were those comparing CBT to waitlist (*g* = − 38).

No significant differences were found between control group type on the size of the effect (Chi^2^ = 4.85, *df* = 2, *P* = 0.09, wait list: *k* = 9, *g* = − 0.53, 95% CI = − 0.97, − 0.09, attention control: *k* = 5, *g* = − 0.00, 95% CI = − 0.18, 0.17, no intervention: *k* = 4, *g* = − 0.10, 95% CI = − 0.44, 0.24). Finally, no significant differences were found based on intervention intensity (delivered weekly, biweekly or twice weekly) on post-test effect size (Chi^2^ = 4.58, *df* = 2, *P* = 0.10; weekly: *k* = 15, *g* = − 0.32, 95% CI = − 0.59, − 0.06; biweekly: *k* = 1, *g* = 0.21, 95% CI = − 0.20, 0.62; twice weekly: *k* = 2, *g* = − 0.18, 95% CI = − 0.48, 0.13).

### Sensitivity analyses

Sensitivity analyses were conducted on studies with a low risk of selection bias, a low risk of attrition bias, and a low risk of contamination (biases deemed most relevant to studies of this nature). Studies with a low risk of contamination had an ES of *g* = 0.03 (*k* = 5, 95% CI = − 0.14, 0.21). The ES for studies with a low risk of selection was *g* = − 0.21 (*k* = 4, 95% CI = − 0.55, 0.13) and for studies with low attrition bias was *g* = − 0.35 (*k* = 11, 61% 95% CI = − 0.65, − 0.05). Funnel plot asymmetry (see Suppl. Fig. 2) suggested a small publication bias [Egger’s test: intercept: − 4.10 [95% CI − 7.59 to 0.61], *t* = 2.49, *P* = 0.02. Results were improved when the Duval and Tweedie’s trim-and-fill method was used to adjust for funnel plot asymmetry (*g* was reduced from − 0.28 to − 0.08). However, this method has been shown to perform poorly where high heterogeneity exists between studies.

## Discussion

Early intervention to reduce symptoms of anxiety is an increasing global priority [56]. To our knowledge, ours is the first review to produce an ES for indicated school-based approaches for child and adolescent anxiety. We have addressed the limitations of previous meta-analyses which focused only on depression [12, 21, 22], mixed settings [12, 15], and/or which merged selective and indicated interventions [15, 17, 20]. We found an overall small post-test ES for anxiety symptom reduction of − 0.28 for intervention groups compared to controls. Large-to-very large effects at post-test were found in 22% of studies (*k* = 4) (− 0.58 to − 2.40) and at follow-up in 46% of studies (*k* = 5) (− 0.41 to − 1.01). Our findings are in line with Neil and Christensen [16]; although their data prohibited a formal meta-analysis, 50% (*k* = 4) of their reviewed trials produced significant small-to-moderate post-test effects on anxiety symptoms ranging from 0.20–0.76. That five (28%) of our reviewed studies had a small but non-significant effect at either post-test or follow-up suggests that lack of power may be masking further potential positive effects.

Our post-test ES on anxiety symptoms is slightly smaller than those reported for universal programs [11–20]. However, concurring with Neil and Christensen [16], our review suggests that indicated programs may be more effective than universal programs over time. By 6 months, and based on nine studies, the post-test ES had increased from − 0.28 to − 0.35, suggesting that young people may take time to benefit from programs. Four studies showed maintenance of a beneficial effect at 6–12 months (− 0.24), but this was no longer evident by > 12 months. This may be because only two studies had sufficient data beyond 12 months, and further positive outcomes may have been missed as the studies with larger, significant post-test ES did not collect long-term outcomes [47, 48]. Good long-term outcomes were also evident in five studies in our review that utilised follow-up diagnostic interviews, and which found that the odds of having an anxiety disorder at 12 and 24 months was lower in the intervention group compared to controls. Whilst this was non-significant at 12 months, the odds became significant at 24 months, suggesting that some indicated prevention programs can prevent the onset of disorder. Thus, whilst there were only a small number of studies collecting long-term data from indicated programs, our review supports Neil and Christensen’s [16] finding that indicated programs can produce good medium-to-long-term outcomes for anxiety reduction in children and adolescents.

Our reviewed studies produced a range of ES at post-test and follow-up. Several factors may be driving this variability, including content type, fidelity of implementation, inclusion of parents and booster sessions, participant engagement, sensitivity of outcome measures and trial quality. We did not find an effect of intervention intensity, although all bar three of our included studies were delivered weekly. Similar to Neil and Christensen [16], we did not find an effect of control group. The absence of an effect of control group suggests that the attention control conditions either did not impact anxiety outcomes or that there was insufficient evidence to detect a difference. Better attention controls, with high therapeutic potential [57], could help to determine whether general vs. specific factors have a role in anxiety prevention.

We could not conduct a sub-group analysis for age, delivery agent, or program type as there were too few studies. Notably, very few interventions were designed to be delivered by teachers. This may be because of the challenges in training teachers in mental health, compared to mental health professionals, for time-limited research studies and/or because of the lack of human and other resources available in schools for delivery by school staff. Findings on delivery agents in general are mixed, from no effect in universal programs [12], to better outcomes from teacher delivered indicated programs for anxiety [16] to better outcomes from mental health professionals for depression programs [20, 21, 58]. Age of participants, complexity of program, delivery support and level of teacher experience and understanding of anxiety vs depression may explain these outcomes.

This meta-analysis should be reviewed in the context of several limitations, including high heterogeneity in study design and methodology and poor study quality. Risk of contamination and publication bias were high. These limitations are common in trials of all program types [19, 20] and high contamination risk suggests that effects may actually be underestimated. Additionally, most studies relied on self-report measures. Although self-report measures do not have sufficient sensitivity and specificity to detect anxiety disorder [59, 60], studies used validated self-report measures that can capture current interfering symptoms, and reduction of scores is meaningful [61]. Some studies in our review involved children with clinical levels of anxiety; we included these studies as the main study population was sub-clinical and the approach was prevention rather than treatment. Most studies reported symptom score cut-offs for inclusion but not upper symptom score cut-offs for exclusion, nor ranges of symptoms scores in the sample. Whilst sample means show that studies largely targeted sub-clinical young people, a minority of young people in the reviewed studies met, or would be likely to have met, clinical thresholds for disorder. Indicated programs may produce even larger effects if only those with high, sub-clinical levels of anxiety (rather than mild-moderate) were recruited. Thus, it is possible that some of the interventions were treatment rather than indicated prevention. However, in the absence of costly pre-screening for program entry, real-world school interventions are likely to attract young people with diverse symptoms. Thus, the reported ES are likely to be ecologically valid.

Future studies should explore the effects of different program intensities and the optimal age for intervention, considering the most critical and plastic periods of development. Although most programs were delivered by MHPs, this is unlikely to be a scalable and sustainable solution given costs and clinician availability. Teacher burden is already high. Evidence from low-and middle-income countries suggests that well-trained lay workers can be effective delivery agents of school mental health programs and future studies in high-income countries should examine their acceptability and effectiveness [62]. Robust assessments of fidelity remain infrequent and should be addressed. Whilst digitised delivery of interventions is growing and could protect fidelity, we need a better understanding of how young people engage digitally, independently, and remotely with mental health content. It is also unclear if and how parental involvement can enhance school-based interventions. Cluster randomisation, attention controls, and detailed descriptions of the allocation sequence generation and concealment would help to improve the quality of future trials of school indicated programs. Future studies should assess outcomes beyond 12 months. As children’s ability to accurately self-report anxiety symptoms may vary considerably across ages, studies should also use multi-informant assessment methods [60]. Adolescents may have different rates of diagnostic accuracy to younger children, but this has yet to be established. Greater use of diagnostic assessments would strengthen the emerging evidence that indicated approaches can prevent anxiety disorder onset. Pragmatic trials in schools are needed to build evidence on implementation, sustainability, effectiveness, and cost-effectiveness.

## Conclusions

Recommendations for implementing school-based interventions should be based on the analysis of multiple factors including intervention cost, the personal and societal burden borne by individuals without intervention, and the likely ES of an intervention [10]. This review of school-based indicated programs for reducing symptoms of anxiety in children and adolescents has produced a favourable post-test ES that exceeds the estimates produced by others for universal prevention programs in schools and in other settings for youth internalising problems [11]. Even stronger effects were found at 6–12 months, which exceeded many ES produced from reviews of universal approaches in school for anxiety symptom reduction. However, the small number of studies with long-term follow-up data, and the considerable heterogeneity and high risk of bias in the reviewed trials means that the findings should be interpreted with caution.

## Electronic supplementary material

Below is the link to the electronic supplementary material.

Supplementary file1 (DOCX 753 kb)
