# Supplementary material for: Indicated prevention interventions for anxiety in children and adolescents: a review and meta-analysis of school-based programs
Source: Eur Child Adolesc Psychiatry. 2020 Jun 13;30(6):849–60. doi: 10.1007/s00787-020-01564-x (PMC8140963; doi:10.1007/s00787-020-01564-x)
Supplement: Supplementary file 1 — Supplementary file1 (DOCX 753 kb) [file 787_2020_1564_MOESM1_ESM.docx]

# Supplementary Material

# Indicated preventative interventions for anxiety in children and adolescents: a review and meta-analysis of school based programs

**Journal:** European Child & Adolescent Psychiatry

**Authors**: Hugh-Jones, S., Beckett, S. & Mallikarjun, P.

**Corresponding author:** Dr Siobhan Hugh-Jones, School of Psychology, University of Leeds, [s.hugh-jones@leeds.ac.uk](mailto:s.hugh-jones@leeds.ac.uk)

# Contents

| Supplementary Table 1 | Search strings by database | Page 2 |
| --- | --- | --- |
| Supplementary Table 2 | Additional details of included trials | Page 5 |
| Supplementary Figure 1 | Risk of bias graph | Page 8 |
| Supplementary Figure 2 | Funnel plot of publication bias for post-test effects on anxiety by sub-groups | Page 8 |

**Table 1: Search strings by database**

| No | PsycINFO | Embase | MEDLINE | CENTRAL |
| --- | --- | --- | --- | --- |
| 1 | (college* or school* or child* or adolescen* or youth).ti,ab | *exp school/* | *exp schools/* | *exp schools/* |
| 2 | *exp anxiety/* | (college* or school* or child* or adolescen* or youth).ti,ab | (college* or school* or child* or adolescen* or youth).ti,ab | *exp school health services/* |
| 3 | *exp anxiety disorders/* | 1 or 2 | 1 or 2 | *exp school nursing/* |
| 4 | (anxiety* or anxious or internalising or internalizing). ti,ab | *exp anxiety/* | *exp anxiety/* | (college* or school* or child* or adolescen* or youth).ti,ab |
| 5 | 2 or 3 or 4 | *exp anxiety disorders/* | *exp anxiety disorders/* | 1 or 2 or 3 or 4 |
| 6 | *primary mental health prevention/ or early intervention/* | (anxiety* or anxious or internalising or internalizing). ti,ab | (anxiety* or anxious or internalising or internalizing). ti,ab | *exp anxiety/* |
| 7 | (anxiety program* or prevent* program* or primary prevention or intervention program* or active intervention* or psychological intervention* or psychological program* or prevent* intervention* or early intervention*). ti,ab | 4 or 5 or 6 | 4 or 5 or 6 | *exp anxiety disorders/* |
| 8 | *online therapy/ or exp psychotherapy/* | *primary prevention/* | *primary prevention/* | (anxiety* or anxious or internalising or internalizing). ti,ab |
| 9 | *exp group psychotherapy/ or group intervention/* | *early medical intervention/* | *early medical intervention/* | 6 or 7 or 8 |
| 10 | *exp counseling/* | (anxiety program* or prevent* program* or primary prevention or intervention program* or active intervention* or psychological intervention* or psychological program* or prevent* intervention* or early intervention*). ti,ab | (anxiety program* or prevent* program* or primary prevention or intervention program* or active intervention* or psychological intervention* or psychological program* or prevent* intervention* or early intervention*). ti,ab | *exp primary prevention/* |
| 11 | *exp behaviour therapy/* | *exp psychotherapy/* | *exp psychotherapy/* | *exp early medical intervention/* |
| 12 | *exp cognitive therapy/ or cognitive behavior therapy/* | *exp group therapy/* | *exp psychotherapy, group/* | (anxiety program* or prevent* program* or primary prevention or intervention program* or active intervention* or psychological intervention* or psychological program* or prevent* intervention* or early intervention*). ti,ab |
| 13 | 6 or 7 or 8 or 9 or 10 or 11 or 12 | *exp counseling/* | *exp counseling/* | *exp psychotherapy/* |
| 14 | (indicat* prevention or indicat* intervention* or indicat* program* or targeted prevention or targeted intervention* or targeted program* or select* prevention or select* intervention* or select* program* or screen* or risk* or vulnerable). ti, ab | *exp behavior therapy/* | *exp behavior therapy/* | *exp psychotherapy, group/* |
| 15 | (RCT* or control* or random* or clinical trial*). ti, ab | *exp cognitive therapy/* | *exp cognitive therapy/* | *exp psychotherapy, brief/* |
| 16 | *exp schools/ or school environnment/ or school facilities/* | *exp cognitive behavioral therapy/* | 8 or 9 or 10 or 11 or 12 or 13 or 14 or 15 | *exp counseling/* |
| 17 | 1 or 16 | 8 or 9 or 10 or 11 or 12 or 13 or 14 or 15 or 16 | (indicat* prevention or indicat* intervention* or indicat* program* or targeted prevention or targeted intervention* or targeted program* or select* prevention or select* intervention* or select* program* or screen* or risk* or vulnerable). ti, ab | *exp behavior therapy/* |
| 18 | 5 and 13 and 14 and 15 and 17 | (indicat* prevention or indicat* intervention* or indicat* program* or targeted prevention or targeted intervention* or targeted program* or select* prevention or select* intervention* or select* program* or screen* or risk* or vulnerable). ti, ab | *exp randomized controlled trials as topic/* | *exp cognitive therapy/* |
| 19 | - | *exp randomized controlled trial (topic)*/* | RCT* or control* or random* or clinical trial*). ti, ab | 10 or 11 or 12 or 13 or 14 or 15 or 16 or 17 or 18 |
| 20 | - | RCT* or control* or random* or clinical trial*). ti, ab | 18 or 19 | (indicat* prevention or indicat* intervention* or indicat* program* or targeted prevention or targeted intervention* or targeted program* or select* prevention or select* intervention* or select* program* or screen* or risk* or vulnerable). ti, ab |
| 21 | - | 19 or 20 | 3 and 7 and 16 and 17 and 20 | *exp randomized controlled trial/* |
| 22 | - | 3 and 7 and 17 and 18 and 21 | - | RCT* or control* or random* or clinical trial*). ti, ab |
| 23 | - | - | - | 21 or 22 |
| 24 | - | - | - | 5 and 9 and 19 and 20 and 23 |

ab = abstract. exp = explode (retrieve results using the selected thesaurus term and all of its more specific terms). ti = title. Thesaurus terms are presented in italics.

Table 2: **Additional details of included trials by date of publication.**

| Trial citation | Country | | Target | Screening Tools | Intervention: Child sessions (#) | Intervention: Parent sessions (#) | Indicators of intervention compliance | Control condition |
| --- | --- | --- | --- | --- | --- | --- | --- | --- |
| Kiselica et al. (1994) | USA | | Anxiety | STAI A-TRAIT | 8 weekly | 0 | No dropout from IG | Active control (skills and guidance classes) |
| Dadds et al. (1997, 1999) | Australia | | Anxiety | RCMAS + teacher nomination + ADIS-P | 10 weekly x 1-2 hr | 3 | Mean attendance  Children = 8.1  Mother = 1.7  Father = 0.63 | No-intervention monitoring group |
| Mifsud & Rapee (2005) | Australia | | Anxiety | RCMAS + teacher nomination | 8 weekly x 1 hr | 2 | 10% (n=5) post-test measure non-completion*** | Waitlist control |
| Bernstein et al. (2005, 2008) | USA | | Anxiety | MASC + Teacher nomination + ADIS C+P | 9 weekly + 2 booster | 9 x 1 hr plus resources | 13.5% (n=5) missed more than 2 CBT sessions | Child only component of FRIENDS program |
| Gillham et al. (2006) | USA | | Anxiety and Depression | RCMAS | 8 weekly x 90 minutes | 6 x 90 minutes | 4.8% (n=1) dropout from IG after 2 sessions | No intervention control condition |
| Siu (2007) | Hong Kong | | Anxiety and Depression | CBCL | 8 weekly | 2 | 34.8% (n=8) missed 1 session | Waitlist control |
| Hunt et al. (2009)* | Australia | | Anxiety | RCMAS + teacher nomination | 10 weekly x 50 mins + 2 booster | 1-2 | Not reported | No intervention control condition |
| Siu (2009) | Hong Kong | | Internalizing problems | CBCL | 8 weekly | 0 | No dropout from IG | Waitlist control |
| Manassis et al. (2010) | Canada | | Anxiety and depression | MASC | 12 weekly x 1 hr | 3 | 3.8% (n=3) dropout from IG | Active control (after school games club + 3 parent sessions ) |
| Liddle & Macmillan (2010)* | Scotland | | Anxiety and Depression | Teacher nomination | 10 weekly | 2 | No dropout from IG | Waitlist control |
| Cooley-Strickland et al. (2011) | USA | | Anxiety | RCMAS + teacher  Nomination + C-DISC | 13 bi weekly | 1 | No dropout from IG | Waitlist control |
| Miller et al. (2011) | Canada | | Anxiety | MASC | 9 weekly | 0 | 4.7% (n=3) post-test non-completion | Attention control (story time) |
| Nobel et al. (2012) | Canada | | Anxiety and Depression | MASC | 12 weekly | 3 | 6.4% (n=5) dropout across both IG and CG but time point not reported | Active control (co-operative and competitive games, after which children ask to reflect on feelings) |
| McLoone & Rapee (2012) | Australia | | Anxiety | SCAS + teacher nomination | 10 weekly | 2 | Average attendance 8.5 / 10 sessions, 16.9% (n=11) post-test measure non completion | Comparator 1 (parent delivery of identical manualised program at home) and Comparator 2 (monitoring only group) |
| Sportel et al. (2013); de Hullu et al. (2017) | The Netherlands | | Anxiety | RCADS + ADIS-C | 10 x weekly 1.5 hr session | 0 | CBT average attendance 6.7/10 sessions. 35% (n=20) dropout from IG. | Comparator 1 (Attention bias modification 20 x 40 min, twice per week online) and Comparator 2 (no intervention control group) |
| Yulei et al. (2015) | | Beijing | Anxiety | SCARED |  | 2 | 1.7% (n=1) post-test measure non completion | No intervention control group |
| Hadwin et al. (2016) | UK | | Anxiety | GA subscale  of SCAS | 5 days per week for 5 weeks (online) | 0 | 35% (n=7) drop-out from IG | Active control (10 x 1hr Friends for Life sessions conducted over 5 weeks. |
| Lam (2016) | Hong Kong | | Anxiety and depression | RCADS + SDQ | 9 weekly |  | 15% (n=3) missed more than 3 of 9 sessions | Waitlist control |
| Scholten et al. (2016) | The Netherlands | | Anxiety | SCAS | 6 (gamed 2 x 1 hr sessions per week over 3 weeks) | 0 | 4.3% (n=3) dropout from IG as too busy | Active control (control game 2 x 1 hr sessions per week over 3 week). |
| Van Starrenburg et al.(2017) | The Netherlands | | Anxiety | SCAS | 12 x 1 hr sessions | 0 | 15.9% (n=10) dropout from IG | Waitlist control |

**Screening measures:** ADIS-C = Anxiety Disorders Interview Schedule – Child; CBCL = Child Behaviour Checklist; C-DISC = Computerised Diagnostic Interview for children; GA = Generalised Anxiety; MASC = Multidimensional Anxiety Scale for Children; RCADS = Revised Children’s Anxiety and Depression scale; RCMAS = Revised Children’s Manifest Anxiety Scale; SCAS = Spence Children’s Anxiety Scale, SCARED = The Screen for Child Anxiety Related Disorders; SDQ = Strengths and Difficulties Questionnaire; SP subscale = Social Phobia subscale; STAI A-TRAIT = State-Trait Anxiety Inventory. **IG** = intervention group; **CG** = control group.

* indicates exclusion from meta-analysis

** dropout calculated from intervention start not intervention allocation

*** post-test is the data collection point at intervention end

**
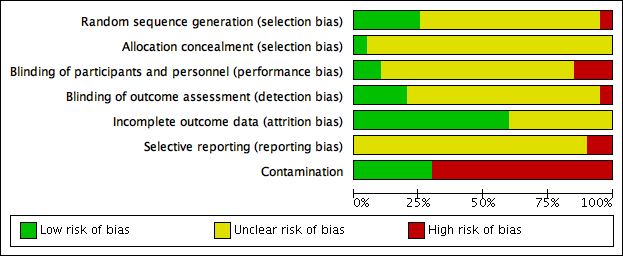
**

**Figure 1**. Risk of bias graph. Percentage of the 20 studies with high, unclear or low risk of bias.


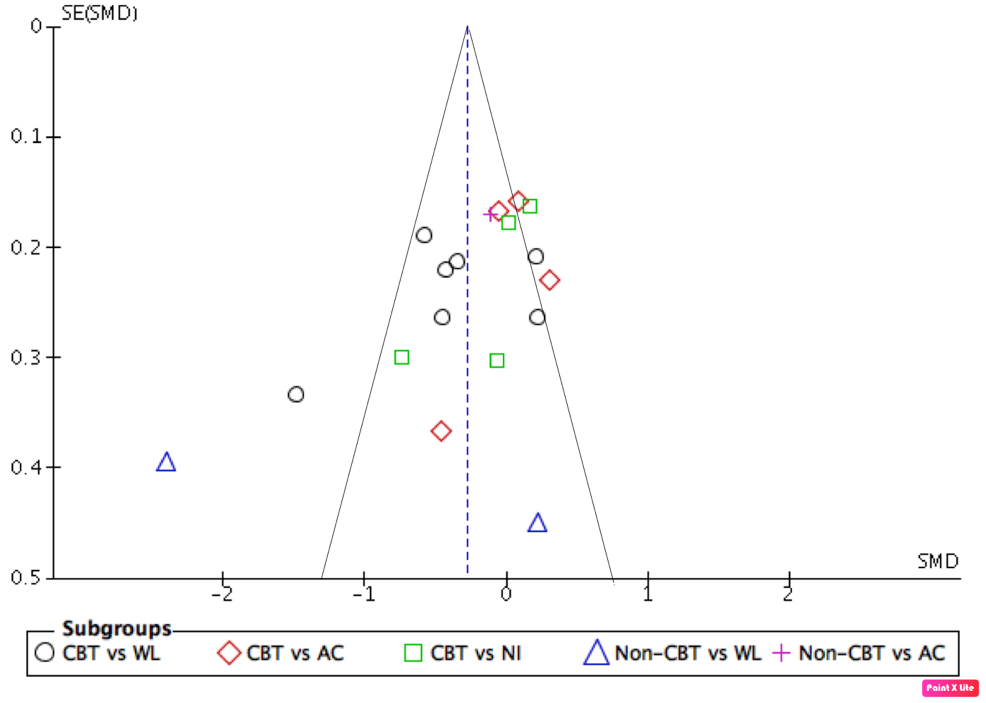


**Figure 2.** Funnel plot of publication bias for post-test effects on anxiety by sub-groups. The x axis shows standard error of standardised mean difference and the y axis is the standardised mean difference. The asymmetry indicates publication bias towards positive findings [Egger’s test: intercept:-4.10 [95% CI,-7.59- 0.61],t = 2.49, P = .02]. Using the trim-and-fill method improved results (Hedges *g* was reduced from -0.28 to -0.08).
